# Real-world effectiveness and safety of direct-acting antivirals for the treatment of hepatitis C virus in kidney and liver transplant recipients: experience of a large transplant center in Brazil

**DOI:** 10.1590/S1678-9946202365059

**Published:** 2023-12-04

**Authors:** Larissa Sgaria Pacheco, Pedro Enrico Ventura, Roger Kist, Valter Duro Garcia, Gisele Meinerz, Cristiane Valle Tovo, Guido Pio Cracco Cantisani, Maria Lucia Zanotelli, Marcos Mucenic, Elizete Keitel

**Affiliations:** 1 Universidade Federal de Ciências da Saúde de Porto Alegre, Programa de Pós-Graduação em Patologia, Porto Alegre, Rio Grande do Sul, Brazil; 2 Irmandade da Santa Casa de Misericórdia de Porto Alegre, Departamento de Nefrologia e Transplante de Rim e Pâncreas, Porto Alegre, Rio Grande do Sul, Brazil.; 3 Irmandade da Santa Casa de Misericórdia de Porto Alegre, Grupo de Transplante Hepático, Porto Alegre, Rio Grande do Sul, Brazil; 4 Universidade Federal de Ciências da Saúde de Porto Alegre, Departamento de Medicina Interna, Porto Alegre, Rio Grande do Sul, Brazil

**Keywords:** Kidney transplant, Liver transplant, Direct-acting antiviral, HCV, Drug interactions

## Abstract

Direct-acting antivirals are the gold-standard treatment for chronic HCV infections, but few studies have investigated their use on kidney and liver transplant recipients. We conducted a real-world study to evaluate the rates of sustained virological response with direct-acting antivirals in kidney and liver transplant recipients. Moreover, it also aimed to evaluate direct-acting antivirals (DAAs) interference with immunosuppressant levels and to describe the frequency of adverse events. As part of this retrospective observational cohort, we included adult patients that had undergone a kidney transplant (KT) or liver transplant (LT) at our center, had a chronic HCV infection, and were treated with DAAs from June 2016 to December 2021. A total of 165 patients were included in the analysis, divided in 108 KT and 57 LT recipients. HCV genotype 1 was more frequent in KT (58.4%), and genotype 3 was more prevalent in LT (57.9%) patients. Sustained virological response was achieved in 89.6% of patients. Adverse effects were reported by 36% of patients. There were significant interactions with immunosuppressants requiring dose adjustments. A total of three episodes of rejection were reported in KT recipients. In conclusion, DAA treatment resulted in high rates of SVR and was well tolerated in both kidney and liver transplant patients. Adverse events were frequent but not severe in most patients, with low treatment drop-out rates. Interactions with immunosuppressants need monitoring since dose adjustments may be required. Reporting real-life experiences is important to help build evidence for patient management in non-controlled environments.

## INTRODUCTION

Hepatitis C virus (HCV) infection remains one of the main causes of chronic liver disease worldwide^1,2^. It is estimated that approximately 58 million people are chronically infected with HCV, with 290,000 HCV-related deaths occurring each year^3^.

Most cohorts of kidney transplant (KT) recipients show that chronic HCV infection is associated with impaired graft function and inferior patient survival, particularly in patients with cirrhosis^4,5^. Specific HCV-related conditions such as glomerulonephritis and an associated increased risk of diabetes can also affect graft outcome^6^.

In liver transplant (LT) recipients, HCV infection is associated with detrimental effects on graft outcomes, increased morbidity, and decreased long-term survival^7^. HCV recurrence is almost universal (>95%) after LT in HCV-infected individuals^8^, and accelerates the disease evolution^9^. Without treatment, 20% of recurrent HCV LT recipients will progress to cirrhosis within five years. Compared to HCV-negative LT recipients, recurrent HCV patients present worse graft function and inferior patient survival^10^.

Thus, effectively and safely managing HCV in kidney and liver transplant recipients is crucial to optimize transplant outcomes. The development of direct-acting antivirals (DAAs) has dramatically changed the HCV treatment scenario, with sustained virological response (SVR) rates of at least 90% in clinical trials and real world experiences^11–13^.

This study explored treatment outcomes in renal and liver transplant recipients with HCV positivity using data from a large transplant center. Our primary objective was to evaluate the rates of sustained virological response with DAAs in this population. Also, we aimed to describe the frequency and relevance of interactions with immunosuppressants, and the frequency and severity of adverse events.

## MATERIALS AND METHODS

### Study design

This is a retrospective cohort study to assess the effectiveness and safety of direct-acting antivirals in the treatment of HCV in kidney and liver transplant recipients from a large transplant center in the Southern Brazil. The study was approved by the local Research Ethics Committee (Nº 4.253.610).

### Subjects

The study sample comprised adult patients subjected to kidney or liver transplant at our center, had a chronic HCV infection, and were treated with DAAs from June 2016 to December 2021, with one year of follow-up.

Chronic HCV infection was defined as the persistence of HCV-RNA for at least six months. Molecular diagnosis of HCV was performed by real-time polymerase chain reaction (PCR) using the Extraction and Amplification Method: COBAS^®^ AmpliPrep / COBAS^®^ TaqMan^®^ HCV Quantitative Test, v2.0 (Roche), for both genotyping and quantification. The lower limit of detection (LLOD) was 15 IU/mL.

The Brazilian Unified Health System (SUS) defined treatment protocols and provided the DAAs, considering HCV genotyping and the presence of cirrhosis. HCV treatment was defined by the hepatologist. The patient was monitored by the hepatologist and the transplant team. Brazilian national guidelines were updated in 2011, 2017, and 2019^14–16^ as new drugs were incorporated. Most regimens were sofosbuvir-based, and combinations included nonstructural proteins 3/4A (NS3/4A) protease inhibitors, NS5A and NS5B polymerase inhibitors.

### Variables

All data were collected from electronic medical charts.

Sustained virological response (SVR12) was defined by a negative or undetectable viral load at or following the 12^th^ week post-treatment.

Adverse events were defined as any clinically relevant medical event that was reported during DAA use. Drug interactions were defined as significant changes of calcineurin inhibitors (CNI) trough levels (30% variation on steady dosage) after treatment initiation.

Baseline characteristics included age, sex, HCV genotype and viral load, primary kidney or liver disease and maintenance immunosuppression. Fibrosis was estimated using two validated scores: 1) Fibrosis-4 Index for Liver Fibrosis (FIB-4) and 2) AST to platelets ratio (APRI) calculators. FIB-4 score was calculated by (Age × AST) / (Platelets × √ALT), and values > 3.25 were considered predictive of advanced fibrosis^17^. The equation for APRI = (AST in IU/L) / (AST Upper Limit of Normal in IU/L) / (Platelets in 10^9^/L), and values ≥ 1.5 were considered highly predictive of significant fibrosis^18^. Transient elastography was not available as a routine. When included, liver stiffness was determined using a Fibroscan machine (EchoSens), reported in kilopascals. Methods are described elsewhere^19^.

Laboratorial results of complete blood count, aminotransferases (AST and ALT), bilirubin, albumin, prothrombin time (PT), gamma-glutamyltransferase (GGT), alkaline phosphatase (AF), glucose, and proteinuria were recorded at baseline, during, and after DAA therapy. Kidney function was evaluated by serum creatinine (mg/dL) and estimated glomerular filtration rate (eGFR) using the CKD-EPI equation at baseline and at 6 and 12 months after treatment.

### Statistics

Categorical variables are presented as numbers and percentages and compared by chi-square and Fisher’s exact test. Continuous variables with normal distributions are presented as mean and standard deviation (SD) and compared by parametric tests. Variables with non-normal distributions are presented as median and 25-75 interquartile and compared by non-parametric tests. The repeated measures analysis of variance (ANOVA) was used to compare means before, during, and after HCV treatment. Significant differences were considered when p < 0.05. All analyses were performed using SPSS^®^ v. 21 (IBM).

## RESULTS

The study included 165 patients, 108 (65.5%) KT and 57 (34.5%) LT recipients. Table 1 shows the baseline characteristics of patients.

**Table 1:** Baseline characteristics of kidney and liver transplant recipients.

Characteristic		Kidney (n= 108)	Liver (n= 57)	p
Age, years (mean ± DP)		50.7 ± 11.1	56.3 ± 8.3	0.052
Male sex, n (%)		62 (57.4)	42 (73.6)	0.043
HCV Genotype, n (%)				< 0.001
	1	63 (58.4)	20 (35.1)	
	2	5 (4.6)	2 (3.5)	
	3	27 (25)	33 (57.9)	
Not recorded		13 (12)	2 (3.5)	
Viral load (log), median (25-75)		6.41 (6.01 - 6.79)	6.22 (5.68 - 6.78)	0.391
Treatment naive, n (%)		93 (86.1)	28 (49.1)	< 0.001
Maintenance immunosuppresion, n (%)				
Tacrolimus		69 (63.8)	40 (70.1)	0.49
Cyclosporine		21 (19.4)	13 (22.8)	0.68
Antiproliferatives		96 (88.8)	20 (35.1)	< 0.001
mTORi		7 (6.4)	21 (36.8)	< 0.001
Fibrosis, n (%)				
Elastography		n = 39 (36.1)	n = 25 (43.8)	0.077
F0	7 (6.4)	5 (8.7)	
F1	10 (9.2)	7 (12.2)	
F2	9 (8.3)	7 (12.2)	
F3	4 (3.7)	2 (3.5)	
F4	9 (8.3)	4 (7)	
APRI > 1.5		20 (18.5)	23 (40.3)	< 0.001
FIB-4 > 3.25		19 (17.6)	23 (40.3)	< 0.001

HCV = Hepatitis C virus; mTORi = mammalian target of rapamycin inhibitors; APRI = aspartate aminotransferase to platelet ratio index; FIB-4 = fibrosis-4 index for liver fibrosis.

For KT recipients, the mean age was 50.7 ± 11.1 years, with male predominance (57.4%). The underlying kidney disease was unknown in 45.4% of patients, hypertension in 7.4%, diabetes in 7.4%, glomerulonephritis in 10.2%, polycystic kidney disease in 10.2%, and others in 19.4% of patients. In KT recipients, fibrosis was estimated by elastography in 39 (36.1%) patients, 13 (12%) had grades F3 and F4. Fibrosis estimated by APRI > 1.5 occurred in 20 (18.5%) and by FIB-4 in 19 (17.6%) patients.

For LT recipients, the mean age was 56.3 ± 8.3 years and there were 42 (73.6%) males. All patients had cirrhosis when transplanted, mostly due to HCV infection (63.2%). HCV plus alcohol was the second most common cause of cirrhosis and occurred in 21 (36.8%) patients. Liver transplant was indicated for HCC in compensated cirrhosis in 27 (47.3%) patients. After transplantation, fibrosis was estimated by elastography in 25 (43.8%) patients, 6 (10.5%) had grades F3 and F4. Fibrosis estimated by APRI > 1.5 occurred in 23 (40.3%) and by FIB-4 in 23 (40.3%) patients.

HCV genotype 1 was detected in 63 (58.4%) KT recipients, and genotype 3 was the most prevalent in LT patients (57.9%).

Maintenance immunosuppression was tailored for each patient, frequently combined with two or more agents. Tacrolimus was the main calcineurin inhibitor (CNI) in KT (63.8%) and LT (70.1%) recipients. Antiproliferatives (mycophenolic acid or azathioprine) were administered to 96 (88.8%) KT and 20 (35.1%) LT recipients. mTOR inhibitors (mTORi) were used mostly in LT recipients (36.8%).

Table 2 shows the different DAA regimens and treatment lengths, defined according to the presence of cirrhosis and HCV genotyping. In total, six (3.6%) patients did not complete the prescribed treatment due to adverse events, five KT recipients and one LT recipients. SVR12 was achieved in 148 (89.6%) of the sample, including 102 (94.4%) KT recipients and 47 (82.4%) LT recipients (p = 0.023).

**Table 2:** Hepatitis C treatment protocols in kidney and liver transplant recipients.

DAA class	Kidney (n = 108)	Liver (n = 57)	p
NS3/4 Inhibitor, n(%)	7 (6.4)	8 (14)	0.15
NS5A Inhibitor, n(%)	104 (96.2)	51 (89.4)	0.09
NS5B Inhibitor, n(%)	103 (95.3)	57 (100)	0.16
Ribavirin, n(%)	21 (19.4)	27 (47.3)	< 0.001
Length of treatment			0.005
1 week*	02 (1.8)	0	
2 weeks*	02 (1.8)	0	
6 weeks*	01 (1.0)	01 (1.8)	
8 weeks	02 (1.8)	0	
12 weeks	99 (91.8)	45 (78.9)	
16 weeks	0	01 (1.8)	
24 weeks	02 (1.8)	10 (17.5)	
Sustained virological response in 12 weeks	102 (94.4)	46 (80.7)	0.023

DAA = direct-acting antivirals; NS = nonstructural proteins.

* treatment abandonment.

**Figure 1:**
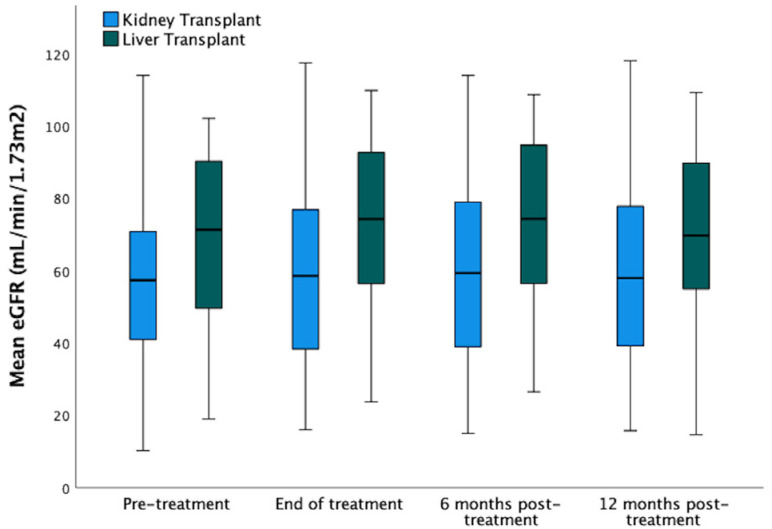
Median of Glomerular Filtration Rate at different times: pre-treatment, end of treatment, 6-month follow-up, and 12-month follow-up. A total of 108 kidney transplant and 57 liver transplant recipients with chronic hepatitis C, treated with direct-acting antivirals, were included. Comparisons were made with repeated measures ANOVA.

Median eGFR did not change after DAA treatment in KT or LT recipients as demonstrated in Figure 1.

**Figure 2:**
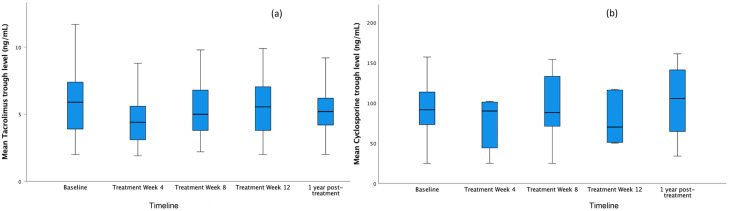
Median blood level of calcineurin inhibitors at different times: before treatment/baseline, week 4, week 8, week 12, and 1-year follow-up. A total of 165 kidney and liver transplant recipients with chronic hepatitis C, treated with direct-acting antivirals, were included. The mean CNI trough levels were significantly decreased (P<0.05) in week 4 compared to baseline: (a) Tacrolimus, (b) Cyclosporine.

During the period of HCV treatment, 60 (36.6%) patients experienced drug interactions with immunosuppressants, either increasing (45%) or decreasing (46.7%) CNI trough levels. In the first four weeks of therapy, the mean CNI trough levels were significantly decreased (Figure 2), and dosing adjustments were necessary in 38 (35.1%) KT and 20 (35%) LT recipients. In total, three (1.8%) patients had to discontinue tacrolimus: two KT recipients had it reintroduced at a lower dose, and one LT recipient had it switched to mycophenolic acid.

A total of 77 (46.6%) patients experienced at least one adverse event (AE) during DAA treatment (Table 3). Anemia was the most frequent, noted in 17 (35.4%) KT and 13 (44.9%) LT recipients. Ribavirin use was related to all cases of anemia, and the drug was suspended in 10 patients and reduced in eight patients. Worsening of kidney function occurred in 11 (14.6%) KT and two (6.9%) LT patients, requiring treatment suspension in four patients.

**Table 3:** Adverse events with direct antiviral agents treatment in kidney and liver transplant recipients.

Adverse event, n (%)	Kidney (n = 108)	Liver (n = 57)	p
None	60 (55.5)	28 (49.1)	0.42
Anemia	17 (35.4)	13 (44.9)	0.29
Other	12 (14.6)	5 (13.8)	0.79
eGFR decrease	11 (14.6)	2 (6.9)	0.22
Neurological	9 (14.6)	6 (13.8)	0.77
Diarrhea	7 (10.4)	2 (6.9)	0.72
Pain	5 (4.2)	4 (6.9)	0.49
Headache	3 (4.2)	1 (3.4)	1.00
Psychiatric	1 (2.0)	2 (6.9)	0.27
Fatigue	0	1 (3.4)	0.34

eGFR = estimated glomerular filtration rate.

Allograft kidney rejection occurred in three patients. Decreasing CNI levels during treatment was noted in one patient, who was treated with methylprednisolone, recovered graft function, and was able to achieve SVR. Another patient had chronic rejection and returned to dialysis during DAA therapy, achieved SVR and received a new kidney transplant shortly after. A different patient had cirrhosis and received 24 weeks of DAA. This patient presented acute rejection afterwards, in a context of low immunosuppression, eventually returning to dialysis.

There were no differences in total bilirubin, albumin, hemoglobin, platelets, INR, urinary albumin creatinine ratio (UACR) at baseline and after 4 weeks, 12 weeks, and 1 year of DAA treatment. There was a significant reduction in the serum concentrations of ALT, AST, alkaline phosphatase, GGT from baseline to week 4, which was sustained up to 1 year post-treatment (Table 4).

**Table 4:** Biochemical results before, during, and after antiviral therapy (DAA) in kidney (n = 108) and liver (n=57) transplant recipients.

	Baseline	4 weeks	12 weeks	1 year	p*
**Total bilirubin** (0.3-1.2 mg/dL)	1.02 ± 0.8	0.73 ± 0.4	0.87 ± 0.6	0.86 ± 0.5	< 0.0001
Kidney	1.02 ± 1.0	0.76 ± 0.3	0.88 ± 0.5	0.98 ± 0.6	0.044
Liver	1.02 ± 0.7	0.71 ± 0.4	0.86 ± 0.7	0.79 ± 0.5	< 0.0001
**AST** (<34 U/L)	69 ± 64.7	31 ± 31.2	32 ± 25.0	30.7 ± 24.2	< 0.0001
Kidney	54.6 ± 50.5	27.4 ± 15.1	28.5 ± 12.6	26.0 ± 9.4	< 0.0001
Liver	104.3 ± 58.6	39.5.4 ± 49.4	43.5 ± 39.4	39.0 ± 37	< 0.0001
**ALT** (10-49 U/L)	73 ± 73.0	31 ± 47.7	31 ± 37.0	28.3 ± 30.9	< 0.0001
Kidney	59.6 ± 65.6	25.0 ± 20.6	23.6 ± 18.0	23.0 ± 14.2	< 0.0001
Liver	113.7 ± 92.4	43.2 ± 79.5	47.7 ± 60.3	39.8 ± 48.6	< 0.0001
**Alkaline phosphatase** (20-130 U/L)	120 ± 66.9	111 ± 58.0	107 ± 62.5	106.4 ± 55.9	< 0.0001
Kidney	102.6 ± 65.4	110.6 ± 54.1	96.2 ± 37.2	98.8 ± 56.3	0.36
Liver	138.1 ± 78.2	116.1 ± 60.8	117.2 ± 78.8	112.5 ± 59.3	0.14
**Albumin** (3.5-5.5 g/dL)	4.1 ± 0.4	4.2 ± 0.4	4.2 ± 0.4	4.2 ± 0.4	0.27
Kidney	4.0 ± 0.5	4.0 ± 0.5	4.1 ± 0.4	4.2 ± 0.5	0.35
Liver	4.2 ± 0.2	4.3 ± 0.2	4.3 ± 0.2	4.3 ± 0.2	0.53
					
**GGT** (males <73 U/L; females < 38 U/L)	234 ± 456.6	66 ± 78.1	88 ± 184.5	94 ± 145.3	< 0,0001
Kidney	139.0 ± 115.7	63.9 ± 59.3	81.5 ± 94.6	67.4 ± 88.6	0.002
Liver	271.3 ± 447.7	61.9 ± 91.2	113.9 ± 260.1	119.6 ± 177.8	0.013
**Hemoglobin** (males 12.8-17.8 g/dL, females 11.6-15.6 g/dL)	13.6 ± 1.8	12.8 ± 2.4	13.3 ± 1.9	13.2 ± 1.9	0.080
Kidney	13.3 ± 2.1	12.5 ± 2.4	12.7 ± 2.0	13.0 ± 1.9	0.002
Liver	13.8 ± 2.2	13.3 ± 2.2	14.1 ± 1.9	13.8 ± 2.0	0.23
**Platelets** (150-440x10^3^/µL)	182.9 ± 66.8	195.9 ± 69.7	187.4 ± 64.4	191.6 ± 66.1	0.010
Kidney	192.1 ± 73.1	201.5 ± 74.4	192.5 ± 57.5	192.6 ± 63.9	0.049
Liver	161.4 ± 53.0	182.1 ± 57.5	174.3 ± 62.6	189.4 ± 71.7	0.006
**INR**	1.09 ± 0.1	1.16 ± 0.3	1.24 ± 0.5	1.16 ± 0.3	0.32
Kidney	1.09 ± 0.1	1.17 ± 0.4	1.22 ± 0.3	1.22 ± 0.3	0.21
Liver	1.09 ± 0.08	1.07 ± 0.07	1.07 ± 0.06	1.07 ± 0.06	0.43
**UACR**	0.66 ± 1.1	0.5 ± 0.5	0.5 ± 0.7	0.6 ± 1.2	0.56
Kidney	0.82 ± 1.4	0.61 ± 0.5	0.53 ± 0.6	0.68 ± 1.2	0.21
Liver	0.56 ± 0.08	0.51 ± 0.01	0.23 ± 0.01	0.18 ± 0.03	0.32
**Glucose** (70-99 mg/dL)	110.0 ± 45.6		105.4 ± 42.0	103.3 ± 36.1	0.10
Kidney	115.5 ± 39.6		101.1 ± 36.8	97.5 ± 27.2	0.21
Liver	125.5 ± 57.2		120.7 ± 50.3	118.1 ± 50.1	0.28

AST = aspartate aminotransferase; ALT = alanine aminotransferase; GGT = gamma-glutamyltransferase; INR = international normalized ratio; UACR = urinary albumin-to-creatinine ratio; normal reference values indicated in parenthesis

* p values comparing baseline to treatment in week 4.

## DISCUSSION

This is one of the largest real-world studies to address the effectiveness and safety of DAAs in chronic HCV treatment in kidney and liver transplant recipients. Our findings reinforce the effectiveness of DAAs in these populations since about 90% of our cohort achieved a sustained virological response. The high effectiveness of DAAs in HCV treatment is consistent with previous studies both on transplant recipients and the general population^11,12,20^.

Despite the positive findings, our study also revealed that almost half of KT and LT recipients experienced adverse events (AE). These figures are higher than those reported by smaller studies. For instance, Fabrizi *et al*.^21^ observed 17.8% of AEs and four suspensions of DAA treatment in KT patients. Silva *et al*.^12^ evaluated 84 LT recipients and observed AEs in a quarter of them, with one DAA therapy discontinued for that cause. A plausible explanation for these differences is the inclusion of ribavirin-containing regimens in our study. Ribavirin use is associated with significant hematological toxicity and drug–drug interactions^22^. In an analysis of 1,952 patients enrolled in phase III ION clinical trials^23^, treatment-related adverse events were observed in 71% of patients that received ribavirin, versus 45% of non-ribavirin therapy, consistent with our results.

The frequent use of ribavirin in this cohort probably relates to greater GT3 prevalence in LT recipients and to previous recommendations of using the drug in immunosuppressed patients, those previously experimented with DAAs and those with advanced liver disease^24^. Previous studies at our center suggested that adding ribavirin to daclatasvir plus sofosbuvir regimen yielded better results, considering that treatment failures occurred only in genotype 3 patients that did not receive ribavirin^25,26^.

The drug interaction of DAAs with immunosuppressants is increasingly clear in the literature. In our study, 36.6% of patients experienced drug interactions with immunosuppressants, requiring dose adjustments. Initial data of HCV treatment with DAA emphasized the substantial risk of drug interactions since Cyclosporin is a moderate inhibitor of CYP3A4 and P-gp, whereas tacrolimus and sirolimus are weak inhibitors of P-gp. Given the narrow therapeutic window of these therapies and individual variability in pharmacokinetics, any change in immunosuppressant levels may lead to toxicity or decreased efficacy^27^.

Plausible explanations for these changes involve an improved hepatic drug metabolism after HCV clearance. This might lead to reduced serum concentrations of CNIs and ultimately may increase the risk of graft rejection. There was an initial significant overall reduction on CNI trough levels by week 4 of DAA treatment, and frequent monitoring allowed dose adjustments to maintain therapeutic levels throughout the follow-up. The European Association for the Study of the Liver^24^ described a decrease in tacrolimus concentrations 12 weeks after DAA therapy. Rejection episodes in our KT recipients was low and comparable to the incidence observed in other studies^28–30^.

Chronic HCV infection causes elevation of liver enzymes, reflecting virus-induced damage to hepatocytes. Viral clearance is frequently accompanied by normalization of these results^31^, including in transplant recipients^12^. In our study, this expected behavior of serum levels of liver enzymes was observed from the 4th week after starting DAA therapy and was maintained throughout the 1-year follow-up.

## CONCLUSION

The main limitation of this study is the retrospective nature. However, we believe it did not compromise the data retrieval from thorough revision of electronic medical charts. The strength of the study is the large number of patients, the inclusion of liver and kidney transplantation, and the long-term follow-up. In conclusion, DAA treatment resulted in high rates of SVR and was well tolerated in both kidney and liver transplant patients. Adverse events were frequent but not severe in most patients, with low drop-out rates. Drug interactions with immunosuppressants need to be monitored and dose adjustments can be required to maintain adequate levels. Reporting real-life experiences is important to help build evidence for patient management in non-controlled environments.
